# Mechanisms underlying the changes in interaction characteristics of beef sarcoplasmic, myofibrillar protein and their correlations induced by ovalbumin and ethanol addition

**DOI:** 10.1016/j.fochx.2026.103905

**Published:** 2026-04-28

**Authors:** Yan Dai, Sha Yi, Zhen Lu, Xiao-li Pan, Yu-xin Li, Yan-yan Chen, Feng Huang, Chun-hong Li

**Affiliations:** a Department of Environmental and Quality Inspection, Chongqing Chemical Industry Vocational College, Chongqing 401228, PR China; b Institute of Food Science and Technology, Chinese Academy of Agricultural Sciences, Beijing 100193, PR China

**Keywords:** Ethanol, Ovalbumin, Characteristics, Sarcoplasmic, Myofibrillar, Beef

## Abstract

This study aimed to preliminarily investigate the effects of 5% ethanol (E) and ovalbumin (OVA) at different concentrations (0.2%–2%) on the interaction characteristics and underlying mechanisms of beef sarcoplasmic proteins (SP), myofibrillar proteins (MP) and their correlations. The results showed that E + 2%OVA group significantly decreased β-sheet% and induced fainter denaturation peaks in SP matrix, which represented degradation and structure changes. Meanwhile, treatments with E + OVAs (>1%) tended to lower gel-related properties, which reflected by the increased proportion of free water (P_22_), coarser and looser gel network in MP matrix. DeoMb%, β-sheet% in SP matrix negatively correlated with hardness, water-holding capacity (WHC), surface hydrophobicity, molecular flexibility, total SH content and P_22_ in MP matrix. This work demonstrated that E + OVAs (≤1%) can improve meat protein-related properties because of mild structural changes and enhanced intermolecular interactions within the SP and MP matrices, thereby improving the meat quality.

## 1 Introduction

Beef, as one of the most nutritious animal-source foods, is popular worldwide owing to its desirable taste, flavor, and high protein content (Ma et al., 2025). Beef sarcoplasmic proteins (SP), including myoglobin (Mb), account for 25%–30% of the total meat protein content, and impact meat colour and water-holding capacity (WHC) (Ma et al., 2025), In contrast, myofibrillar proteins (MP), which constitute approximately 50%–60% of the total meat protein content, are closely associated with the textural and structural properties of beef and its products (Rao et al., 2024). Additionally, beef protein can also promote muscle protein synthesis and increase total iron intake, thereby theoretically benefiting both young and elderly individuals (Valenzuela, Mata, Morales, Castillo-García, & Lucia, 2019).

Protein-protein interaction (PPI) and protein-alcohol interaction have numerous formulation applications through structure modification of protein complexes, thus promoting stability, functionality and quality in food, nutraceutical, and pharmaceutical matrices (Alrosan, Tan, Easa, Gammoh, & Alu'datt, 2022; Liu et al., 2023). The PPI (exogenous addition or specific physical contacts of two or more proteins) is steered by non-covalent forces and often occurs in a cell-type, condition-, and organism-specific manner (Jia & Wu, 2023). The dynamic balance of release and retention of polar flavor compounds such as alcohols can also interact with protein-based systems through the formation of hydrogen bonds, largely affecting the sensory and consumer acceptability of food (Qian et al., 2024). Ovalbumin (OVA), accounting for approximately 54% of the total egg white protein (EWP), can be widely utilized in animal or plant protein-based food system, improving nutritional, sensory and techno-functional properties through PPI (Yu et al., 2024). The acyl-modified OVA-MP gels, treated by CaCl_2_ (0.30 mol/L) were markedly enhanced (Wu et al., 2025). Yu et al. (2024) demonstrated that the addition of 0.5% phosphorylated-OVA (P-OVA) could effectively enhance MP gel properties through higher zeta potential and exposed hydrophobic interactions. Texture properties of 3D-printed mycoprotein (MYC) meat alternatives with 6% EWP were comparable to beef (Muhedaner et al., 2025). Low supplemental level (the ratio of 16: 1 w/w) of EWP could inhibit the rapid aggregation of MP, thus prolonging the formation time of 3D network structure of protein, and forming a more orderly “cage” structure (Niu et al., 2025).

Various alcohols have the capacity to interact with proteins, thus altering the structure and functional properties of proteins. The polyhydroxy alcohol-mediated curing including 4% NaCl, 1% glycerol, 1% xylitol and 1% sorbitol can interfere with the exposure of hydrophobic groups, making the MP structure tighter and altering the functional properties of MP (Liu et al., 2023). 1,2-Propanediol (3%) and glycerol (5%) enhanced the gel properties of sliver carp by inducing desirable unfolding and aggregation of myosin molecules (Xiong et al., 2025). The evolution of electrostatic repulsion inside the molecule caused by the utilization of ethanol (0.3%–1.5%, v/v) was the main reason for the changes in the physical properties of chicken MP (CMP) solutions and gels (Zhou et al., 2019). The edible insect *Tenebrio molitor* protein (ETMP) treated with 20% ethanol, can be utilized as an alternative protein source and functional food because of the changes in techno-functional properties (Lee, Kim, Kim, Song, & Choi, 2024). Ethanol (10%) may induce the preferential unfolding of silver carp myosin heads, leading to the aggregation of myosin molecules in a head-to-head fashion (Xiong et al., 2024).

It is also well established that wine and EWP (including OVA) addition can significantly influence quality properties of meat products (Dai et al., 2023; Gao et al., 2024). The rice wine lees, that applied to the ham surface, also effectively promoted proteolysis and the formation of taste-active compounds of traditional dry-cured hams (Ding et al., 2026). Currently, extensive studies based on the interactions among meat protein, ovalbumin and ethanol are mainly focused on limited gelation and flavor properties.

However, the internal mechanism of OVA and ethanol addition in regulating the interactions, correlations of SP and MP system, and meat quality attributes has still not been sufficiently explained. In addition, the interactive effects on the structural and functional properties, and the internal correlations between SP and MP, treated by ethanol and ovalbumin are still limited. Therefore, this study preliminarily prepared the meat SP and MP models, respectively, and mainly analyzed the influence of OVA and ethanol addition on the interactions between SP and MP in models, thus better understanding the changes in meat quality during processing.

We hypothesized that the structural and functional properties in SP and MP might be regulated and have some internal correlations via the addition of ethanol and ovalbumin. To test this hypothesis, we investigated the generation pattern of ovalbumin and ethanol addition in beef SP and MP systems respectively, thus determining the regulation in structural and functional properties of protein complex, as well as exploring the correlations between SP and MP. This research also contributes to a deeper understanding of the mechanism of OVA-ethanol-based meat protein complexes, which is essential for the future development of meat products with improved quality attributes.

## 2 Materials and methods

### 2.1 Materials

In this study, the fresh beef tenderloin (*psoas Major*, PM) muscle samples (3 kg) from four Chinese Simmental cattle (18–20 months old, 326–376 kg live weight) were supplied by a local commercial abattoir of Chongqing Zhenghe Agricultural Technology Co., Ltd. (Chongqing, China). The samples were taken from the carcass at 24 h postmortem at 4 °C, and immediately transported to the laboratory of Chongqing Chemical Industry Vocational College on the following day for subsequent treatments within 30 min. Ovalbumin powder (OVA, purity≥80%), absolute ethanol, 5,5′-dithiobis-(2-nitrobenzoic acid) (DTNB), 2,4-dinitrophenyl hydrazine (DNPH) and phenylmethanesulfonyl fluoride (PMSF) were purchased from Shanghai Yuanye Biotechnology Co., Ltd. (Shanghai, China). A standard protein marker (11–245 kDa) was purchased from Shanghai Macklin Biochemical Co., Ltd. (Shanghai, China). All other chemicals were of analytical grade.

### 2.2 Extraction of SP and MP

The extraction of SP was performed with reference to previous reports (Dai et al., 2013). Briefly, fresh beef tenderloin (5 g) was minced and mixed with 15 mL of extraction buffer (pH 7.6) containing 20 mM Tris, 2 mM ethylenediaminetetraacetic acid disodium salt (EDTA-2Na), and 10 μL/L PMSF. The mixture was homogenized at 11,000 rpm for 30 s and then centrifuged at 10,000×*g* for 10 min. The supernatant was collected as the SP extract.

The resulting precipitation (myofibrils) was washed three times with 50 mM KCl buffer (pH 7.4), and then resuspended in 15 mL of the same KCl solution for MP extraction. The protein concentrations of SP and MP were determined using the biuret method and expressed as mg protein/g meat (Dai et al., 2023).

### 2.3 Establishment of SP and MP systems

The SP and MP systems were constructed according to previous reports with some modifications (Dai et al., 2023; Yu et al., 2024; Zhou et al., 2019). Briefly, predetermined amounts of ethanol (0%, 5%, v/v) and OVA (0%–2%, m/v) was dissolved in SP and MP solutions respectively, under constant stirring for 2 h until further analysis (Table 1).

**Table 1:** The formulations of SP and MP solution with varying percentages of ethanol and OVA.

Treatments (SP and MP)	Materials	
	absolute ethanol (*v*/*v*)	OVA (*m*/*v*)
Control	0%	0%
E	5%	0%
E + 0.2%OVA	5%	0.2%
E + 0.6%OVA	5%	0.6%
E + 1%OVA	5%	1%
E + 1.4%OVA	5%	1.4%
E + 2%OVA	5%	2%

SP, sarcoplasmic protein; MP, myofibrillar protein; OVA, ovalbumin; Control, the untreated group; E, absolute ethanol.

### 2.4 Sodium dodecylsulfate polyacrylamide gel electrophoresis (SDS-PAGE)

After adjusting the SP and MP solutions to the same protein concentration (2 mg/mL), all samples (including the standard protein marker) were mixed with loading buffer (2% SDS, 10% glycerol, 0.1 M Tris-HCl (pH 6.8), 1% β-mercaptoethanol, a trace of 0.05% bromophenol blue) at a ratio of 1:1, The mixtures were boiled for 3 min and then subjected to SDS-PAGE using an electrophoresis system (DYCZ-MINI, Beijing Liuyi Biotechnology Co., Ltd., China, Beijing) with 12% polyacrylamide resolving gel and a 4% stacking gel at a constant current (30 mA) for 1.5 h (Dai et al., 2013). The gels were stained in 1 g/L Coomassie Brilliant Blue R-250, distained in a solution containing 120 mL/L methanol and 75 mL/L acetic acid, placed on a light board and photographed using a digital camera.

### 2.5 Confirmation of beef SP characteristics

#### 2.5.1 CD spectral analysis

Circular dichroism (CD) spectroscopy was performed to analyze the secondary structure content of beef SP according to Jiang, Xia, Gong, Ma, and Sun (2024). A beef SP solution (1 mg/mL) was used for spectral recording over a wavelength range of 190–250 nm using a CD spectroscopy (MOS-500, BioLogic, Seyssinet-Pariset, AuRA, France, Paris) with a 0.1 cm thick CD cuvette at 25 °C. The results were calculated using CDNN software.

#### 2.5.2 Determination of proportions of Mb

The relative contents and forms of Mb were determined following the calculation method described by Dai et al. (2013). The absorbance was measured separately at wavelengths of 525, 545, 565, and 572 nm using a UV–Vis spectrophotometer (Puxi T6, Beijing Puxi general instrument Co., Ltd., China, Beijing).

#### 2.5.3 Differential scanning calorimeter (DSC)

After extraction, all beef SP samples were lyophilized using a lyophilizer (SCIENTZ-10 N, Ningbo Scientz Biotechnology Co., Ltd., China, Ningbo). Subsequently, DSC experiments were performed using a DSC instrument (DSC 8000, PerkinElmer, USA, New York) according to the method described by Rao et al. (2024). Briefly, 5 mg of each sample was mixed with 1 μL of distilled water, weighed into aluminium pans, and heated at a rate of 10 °C/min over a temperature range of 20 °C to 90 °C. The protein thermal denaturation temperature (*T*_max_) and enthalpy of absorption (J/g) were analyzed using the software provided with the instrument.

### 2.6 Confirmation of beef MP characteristics

#### 2.6.1 Endogenous intrinsic fluorescence (EIF)

The EIF spectra were acquired following a previously reported method (Song, Zhang, Qi, Yin, & Wang, 2024). Briefly, the fluorescence spectra of beef MP samples (0.1 mg/mL) were determined using a fluorophotometer (Shimadzu-F-700098, Shimadzu (Shanghai) Global Laboratory Consumables Co., Ltd., China, Shanghai). The instrument parameters were set as follows: excitation wavelength was 283 nm, emission spectral range was 300–400 nm, and scanning speed was 240 nm/min.

#### 2.6.2 Protein surface hydrophobicity and molecular flexibility

Surface hydrophobicity was measured using the method described by Yu et al. (2024) with minor modifications. One milliliter of MP sample (1 mg/mL) and the control (without MP) were mixed thoroughly with 200 μL of bromophenol blue (BPB) solution (1 mg/mL). After centrifugation at 7500×*g* for 15 min, the supernatant was diluted 10-fold with distilled water, and the absorbance values (*A*_sample_, *A*_control_) were measured at 595 nm using a UV–Vis spectrophotometer (Puxi T6, Beijing Puxi general instrument Co., LTD, China, Beijing). The amount of BPB bound per milligram of protein was calculated using the following formula:

$$\mathrm{BPB}\;\text{bound}\;\left(\mathrm{\mu g}\right)=40\;\mathrm{\mu g}\;\mathrm{BPB}\times \left(A_{\text{control}}-A_{\text{sample}}\right)/A_{\text{control}}$$

Molecular flexibility was determined as previously described by Yang et al. (2024). Briefly, 250 μL of trypsin solution (1 mg/mL) was mixed with 4 mL of MP solution (1 mg/mL), heated in a water bath at 38 °C for 5 min, and the reaction was terminated by adding 4 mL of 5.0% trichloroacetic acid (TCA). The mixture was centrifuged at 4000 ×*g* for 30 min, and the absorbance of the supernatant was measured at 280 nm using a UV–Vis spectrophotometer (Puxi T6, Beijing Puxi general instrument Co., Ltd., China, Beijing).

#### 2.6.3 Protein oxidation

Carbonyl content was measured according to the method of Dai et al. (2023). Briefly, 500 μL of MP solution was mixed with 2 mL of 10 mM DNPH in 2.0 M HCl, incubated in the dark for 1 h, followed by the addition of 2.0 mL TCA (20%, m/v), and centrifuged at 8000×g for 10 min. Subsequently, the residue was collected and washed with ethyl acetate/ethanol (1:1 v/v) until colorless. The absorbance at 370 nm was recorded using a a UV–Vis spectrophotometer (Puxi T6, Beijing Puxi general instrument Co., Ltd., China, Beijing) after the addition of 6.0 M guanidine hydrochloride in 20 mM potassium phosphate buffer (pH 6.5) (3 mL) to calculate carbonyl content using an absorption coefficient of 2.2 × 10^4^ M^−1^·cm^−1^.

Total sulfhydryl (SH) groups of all MP solution was measured according to the procedure described by Song et al. (2024). Briefly, the MP solution was diluted to 5 mg/mL with 20 mmol/L Tris-HCl (containing 0.6 mol/L NaCl, pH 7.2). The diluted protein solution (1 mL) was added to the test tube, followed by 9 mL of 50 mmol/L phosphate buffer (0.6 mol/L KCl, 10 mmol/L EDTA, 8 mol/L urea, pH 7.0) and 0.5 mL of 0.1% DTNB (m/v, prepared with 50 mmol/L phosphate buffer, pH 7.0). The mixture was reacted in a water bath at 25 °C for 25 min, and the absorbance of total SH groups was measured at 412 nm using a UV–Vis spectrophotometer (Puxi T6, Beijing Puxi general instrument Co., Ltd., China, Beijing) with an absorption coefficient of 13,600 L·(mol·cm)^−1^.

#### 2.6.4 Protein particle size and zeta (ζ) potential

Particle size and *ζ* potential of MP solutions were determined using a laser particle size analyzer (Zetasizer Nano ZS ZEN3600, Malvern Instruments Ltd., UK, London) via dynamic light scattering (DLS) at 25 °C according to Huang et al. (2022). Particle size determination: The MP solution (1 mg/mL, 1.5 mL) was added to a cuvette for particle size determination. *ζ* potential determination: The MP solution (1 mg/mL, 0.6 mL) was added to a cuvette for *ζ* potential value of the sample.

#### 2.6.5 Rheological behaviors

The rheology behaviors of raw MP-containing precipitations were determined according to the method of Zhou et al. (2019) with some modifications. Tests were carried out with a PP50 parallel plate (50 mm diameter) at 25 °C using a rheometer (MCR 301, Anton Paar (Shanghai) Trading Co., Ltd., China, Shanghai). The MP samples were placed between two parallel plates with an interval (1 mm), equilibrated at 20 °C for 10 min, heated from 20 °C to 80 °C at a rate of 2 °C/min and held at 80 °C for 10 min. The dynamic frequency was arranged from 0.1 to 20 Hz with a strain of 1.0%. The storage modulus (*G*'), loss modulus (*G*") and phase angle tangent (tan δ) were automatically recorded.

#### 2.6.6 Determination of MP gel properties

MP gels were prepared by centrifuging MP-containing residues at 9000×*g* for 10 min, heating in a water bath at 90 °C for 30 min, and cooling to room temperature for gelation analysis. The textural properties (hardness, springiness, cohesion, gumminess and chewiness) of MP gels were measured by a texture analyzer with a P/36r probe attached to a texture analyzer (TA.TX2, Stable Micro Systems Ltd., UK, Surrey) according to the method of Dai et al. (2023).

The water-holding capacity (WHC) of MP gels was determined according to the method of Yu et al. (2024). Briefly, the gels (3 g) along with filter paper were placed into 50 mL centrifuge tubes, centrifuged at 9000×*g* for 20 min. The WHC was calculated as the ratio of mass of gel after centrifugation to the mass of gel before centrifugation.

The water distribution of MP gels (2 g) was weighed, placed in cylindrical glass tubes with a diameter of 15 mm, and measured according to the method of Zhang et al. (2025) using a LF-NMR analyzer (VTMR20–010-T, Suzhou Niumag Analytical Instrument Corporation., China, Suzhou) operated at 23 MHz and 0.5 Tesla of magnetic field strength. The transverse relaxation times (*T*_2_), including *T*_2b_ (bound water), *T*_21_ (immobilized water), and *T*_22_ (free water) and their corresponding area fractions (*P*_2_), including P_2b_, P_21_, and P_22_ were calculated by Carr-Purcell-Meiboom-Gill (CPMG) sequence.

All MP gels were lyophilized by a lyophilizer (SCIENTZ-10N, Ningbo Scientz Biotechnology Co., Ltd., China, Ningbo) for Fourier transform infrared (FTIR) spectroscopy and morphological analysis. The secondary structure of MP gels was analyzed using a FTIR spectrometer (ART-TENSOR 27, Buruker, German, Berlin) according to the method of Yu et al. (2024). The transmittance spectra were recorded over a wavelength range of 4000–500 cm^−1^ with a resolution of 4.0 cm^−1^.

The morphology of MP gels was observed using a scanning electron microscopy (SEM, Zeiss Gemini Sigma300, Shenzhen Blue Starry Electronic Technology Co., Ltd., Shenzhen) after platinum sputtering of the samples. Observations were conducted under high vacuum at 3.0 kV with a magnification of 18,000 × .

### 2.7 Statistical analysis

All experiments, except for SDS-PAGE, DSC, EIF, rheology and SEM, were performed in triplicate. The results were expressed as mean ± standard errors (SE) and analyzed using SPSS (version 16, IBM, USA, New York). One-way analysis of variance (ANOVA) and Duncan's multiple range test were employed to determine significantly differences (*P* < 0.05) in all parameters of beef protein systems, excluding those related to SDS-PAGE, DSC, EIF, rheological behaviors and SEM. Pearson correlation analysis and other graphs were conducted using Origin (2021, OriginLab, USA, New York).

## 3 Results and discussion

### 3.1 Protein content and SDS-PAGE analyses of SP and MP

**Fig. 1:**
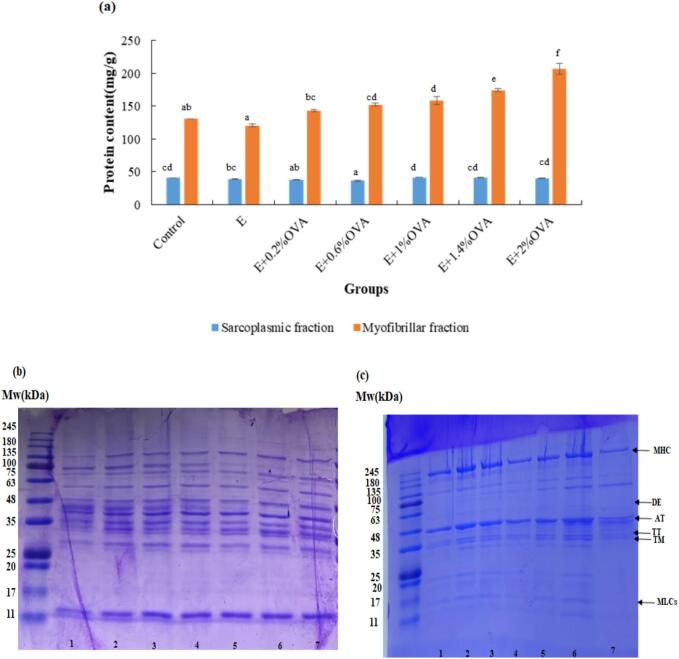
The protein content of beef (a) SP and MP, SDS-PAPE patterns of (b) SP and (c) MP fractions. Abbreviations: Control, the untreated group; E, absolute ethanol; OVA, ovalbumin; 1, Control; 2, E; 3, E + 0.2%OVA; 4, E + 0.6%OVA; 5, E + 1%OVA; 6, E + 1.4%OVA; 7, E + 2%OVA; MW, protein standard; MHC, myosin heavy chain; DE, desmin; AT, actin; TT, troponin-T; TM, tropomyosin; MLCs, myosin light chains; Values are means ± SE of three replicates measurements. Error bars represent positive standard errors of the mean. Different lowercase letters (a-f) donate significant difference in these treatment group samples (*P* < 0.05). Means having no letter do not significantly differ at *P* < 0.05.

As shown in Fig. 1(a), the content of beef SP fraction in the E + 0.2%OVA and E + 0.6% OVA groups (36.72, 37.93 mg/g, respectively) was significantly (*P* < 0.05) lower compared to those of the control, E + OVA (1%, 1.4%, 2%) groups (40.03–41.73 mg/g). Simultaneously, compared with those of the control and E groups, the content of beef MP fraction increased drastically in E + OVA (0.6%, 1%, 1.4%, 2%) groups. The interaction between E and OVA might induce the precipitation of SP, thereby increasing MP content in accordance with Liu, Ruusunen, Puolanne, and Ertbjerg (2014). Changes in OVA concentration may not only affect the total extractability of meat proteins, but also modify the composition and relative distribution of SP and MP system, thereby influencing the functionality (Yang, Xiong, & Jiang, 2023).

Based on the SDS-PAGE results (Fig. 1(b)), protein bands with molecular weights of 63–75 kDa and 35–48 kDa disappeared in the beef SP fraction of E + OVA(1%, 1.4%, 2%) groups, indicating slightly higher protein degradation according to Liu et al. (2024). The SP bands (20–63 kDa) were also detected. Notably, slightly intense bands with molecular weights of 100 kDa, 25–35 kDa, and 11–17 kDa were identified in the E and E + OVA groups, which probably resulted from SP precipitation, leading to increased MP content.

All MP samples displayed typical protein patterns, including myosin heavy chain (MHC), desmin (DE), actin (AT), troponin-T (TT), tropomyosin (TM) and myosin light chains (MLCs), which was consistent with a previous study (Song et al., 2024). A corresponding increase in the band intensity of the beef MP fraction (75–100 kDa) was observed in the E + OVA (1%, 1.4%, 2%) groups. The intensity of MP protein bands (17–20, 48–63, 100–135 kDa) weakened markedly in the E + OVA(0.6%, 1%, 2%) groups. The MP samples treated by E and E + 0.2%OVA showed a remarkable increase in the intensity of MHC, AT, TM and MLCs bands relative to that of the E + 2%OVA group. This indicated that higher doses of OVA (>1%) might facilitate protein cross-linking, aggregation and polymerization in MP-ethanol matrix, which is in agreement with the observations of Li, Wu, Tang, and Wang (2024). However, there were still limits that not all high-molecular-weight proteins were shown in this SDS-PAGE figure by applying 12% resolving gel.

### 3.2 CD spectra, Mb relative content and DSC analysis of SP

**Fig. 2:**
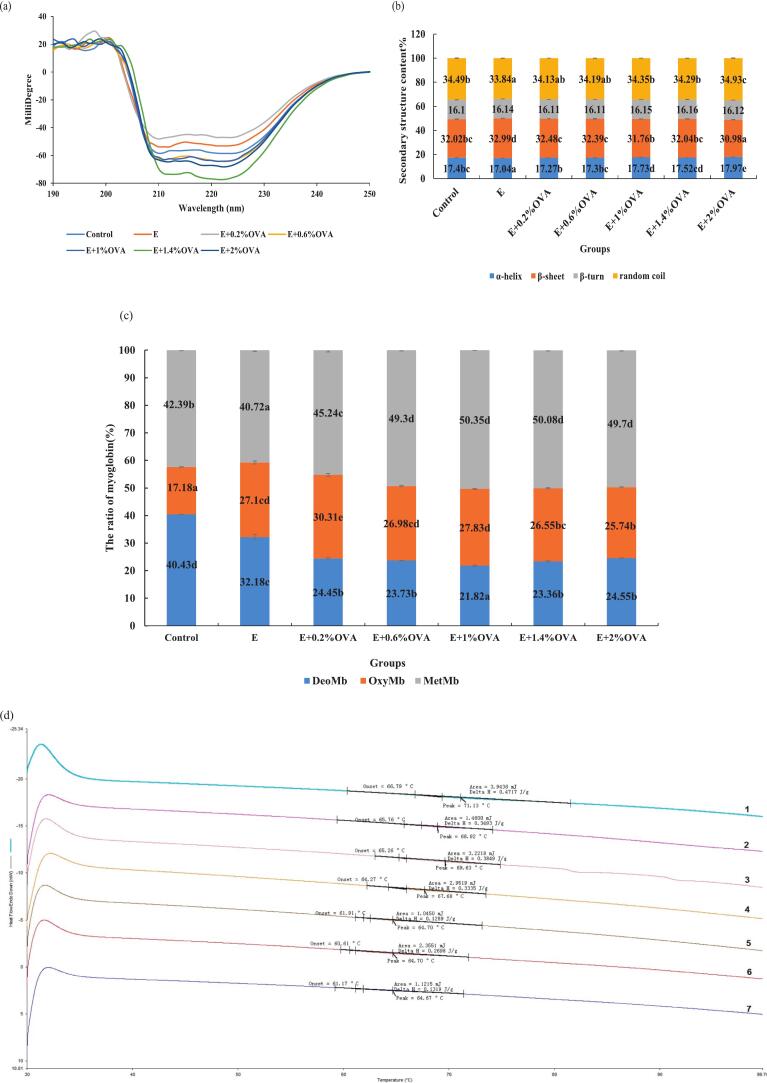
Changes in (a) CD spectrum, (b) secondary structure content (%) of SP fractions, (c) Mb forms and relative content (%) and (d) DSC thermogram of SP fractions in all group beef. Abbreviations: Control, the untreated group; E, absolute ethanol; OVA, ovalbumin; Mb, myoglobin; CD, circular dichroism; DSC, differential scanning calorimetry; DeoMb, deoxymyoglobin; OxyMb, oxymyoglobin; MetMb, metmyoglobin; 1, Control; 2, E; 3, E + 0.2%OVA; 4, E + 0.6%OVA; 5, E + 1%OVA; 6, E + 1.4%OVA; 7, E + 2%OVA. Values are means ± SE of three replicates measurements. Error bars represent positive standard errors of the mean. Different lowercase letters (a-f) donate significant difference in these treatment group samples (*P* < 0.05). Means having no letter do not significantly differ at *P* < 0.05.

The CD spectra of all beef SP fractions are shown in Fig. 2(a), and the contents of α-helix, β-sheet, β-turn and random coil are presented in Fig. 2(b). Among all groups, the E + 2%OVA group exhibited the highest α-helix percentage and random coil percentage, while it had the lowest β-sheet percentage (*P* < 0.05). This phenomenon could be attributed to the disruption of the secondary structure of SP during degradation (Jiang et al., 2024). No significant difference in β-turn% was detected among all beef SP groups (*P* > 0.05).

Myoglobin (Mb) exists in three forms (metmyoglobin (MetMb), oxymyoglobin (OxyMb), and deoxymyoglobin (DeoMb)), which reflect the stability of Mb and meat colour changes (Dai et al., 2013). Additionally, Mb can induce structural changes, oxidative aggregation and gel degradation in MP (Guan et al., 2025). As shown in Fig. 2(c), a decrease of DeoMb% and an increase in MetMb% were observed, particularly in the beef SP fractions of the E + OVA treated groups. The control group showed the highest DeoMb% (40.43%), whereas the E + 1%OVA group had the lowest DeoMb% (21.82%). The MetMb% sharply increased (*P* < 0.05) in the E + OVA (0.6%, 1%, 1.4%, 2%) groups. Moreover, the OxyMb% in the E + OVA (0.2%, 1%) groups was significantly (*P* < 0.05) higher than those of the control, E + OVA (1.4%, 2%) groups. The ethanol and OVA may alter the dynamic balance in the Mb redox system, facilitating the oxidation of DeoMb, and its rapid conversion into MetMb (Jiang et al., 2024). A similar finding was also reported by Zhao, Zhu, and Wang (2024) in their study on lactic acid-treated yak meat.

Fig. 2(d) displays the DSC thermogram of beef SP fractions in all 7 groups. The extracted beef SP exhibited a single, weak thermal absorption peak with transition temperature (*T*_max_) ranging from 64.67 to 71.13 °C and an enthalpy (ΔH) ranging from 0.1299 to 0.4717 J/g. The identified peak apex temperature of beef SP was closely consistent with that of raw lamb (Rao et al., 2024). The decreasing values of *T*_max_ in the E and all E + OVA groups indicated that ethanol alone or in combination with OVA could impair the structural stability of SP, generating SP-based macrolecular polymers with disordered structure. The oxidation and degradation processes might occur in the E, E + OVA treated groups, which destabilized the SP system and thereby reduced its thermal stability (Li et al., 2024). The slightly fainter SP denaturation peaks in especially E + OVA (1%–2%) samples suggested that excessive OVA addition (>1%) might have resulted in SP removal and modifications to protein composition and structural organization (Marchetti, Perez, Czerner, & Loredo, 2025).

### 3.3 EIF, surface hydrophobicity and molecular flexibility of MP

**Fig. 3:**
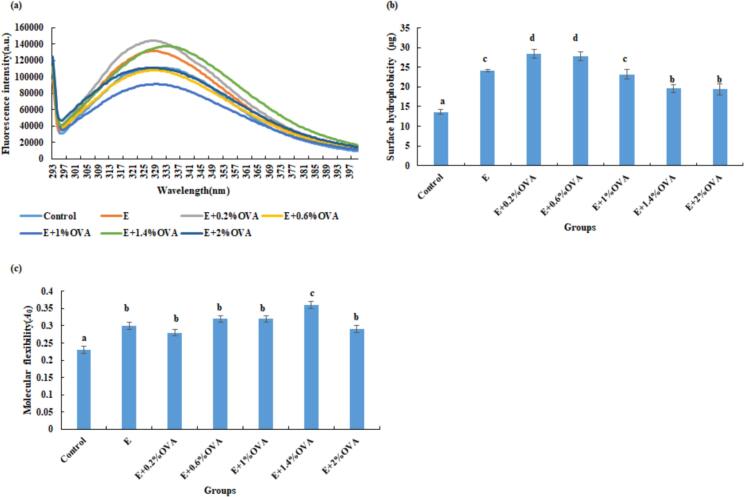
Changes in (a) endogenous fluorescence spectra, (b) surface hydrophobicity, and (c) molecular flexibility of MP fractions in all group beef. Abbreviations: Control, the untreated group; E, absolute ethanol; OVA, ovalbumin. Values are means ± SE of three replicates measurements. Error bars represent positive standard errors of the mean. Different lowercase letters (a-f) donate significant difference in these treatment group samples (*P* < 0.05). Means having no letter do not significantly differ at *P* < 0.05.

The EIF spectra of beef MP fractions can characterize the tertiary structure of proteins, as tryptophan (Trp) and tyrosine (Tyr) residues are sensitive to the polarity of the microenvironment (Xu et al., 2023). As shown in Fig. 3(a), the fluorescence intensity of MP was slightly stronger in the E and E + OVA (0.2%, 1.4%) groups than in the other 4 groups. Moreover, the maximum emission wavelength of the E + 1.4%OVA group MP exhibited a red shift, which was attributed to the strengthening of O···H hydrogen bonds at the expense of amide C=O bond, weakening the hydrophobicity of amino acid residues of MP (Herberhold et al., 2003; Xu et al., 2023). This result suggests that treatments with ethanol along, or ethanol combined with OVA (0.2%, 1.4%) can prevent the unfolding of the MP structure, maintaining MP in a more folded state due to the enclosed and enhanced polarity of Trp and Tyr residues (Xu et al., 2023). This is also consistent with the findings of Liu et al. (2023), who reported that MP treated with polyhydroxy alcohols exhibited higher fluorescence intensity. It was also demonstrated that the ethanol, similar to β-sanshool, might bind to MP, subsequently inhibiting the cleavage of chemical bonds and the degradation of endogenous amino acids (Xu et al., 2023). The decrease fluorescence intensity of MP in the other groups might be attributed to the exposure and oxidative modification of tryptophan residues (Cheng et al., 2025).

In contrast to that of the control group (13.66 μg), the surface hydrophobicity of MP increased significantly (*P* < 0.05) in the E and E + OVA groups with values ranging from 19.39 to 28.31 μg. Meanwhile, the surface hydrophobicity in the E and E + OVA groups first increased from 24.10 μg to 28.31 μg and then decreased to 19.39 μg (Fig. 3(b)). Treatments with E alone and in combination with OVA may disrupt the balance between external hydrophilic interactions and internal hydrophobic interactions of MP, which is associated with intermolecular aggregation and cross-linking. However, excessive OVA addition (>1%) might further mask the exposed surface hydrophobic groups, resulting in reduced surface hydrophobicity. A similar trend was also observed in konjac glucomannan and β-sanshool treated MP (Gao et al., 2022; Xu et al., 2023).

The molecular flexibility of MP in the E, E + OVA groups (0.28–0.36) was significantly (*P* < 0.05) higher than that of the control (0.23), with the highest value observed in the E + 1.4%OVA group (Fig. 3(c)). Higher molecular flexibility might modify MP, leading to easier structural rearrangement and adsorption, as well as improved emulsifying and surface properties (Cui et al., 2020). Yang et al. (2024) reported a similar increase in the molecular flexibility of ultrasound (UT)-treated soy protein isolates and MP extracted from low-salt meat batters under UT treatment.

### 3.4 Protein oxidation, particle size and ζ potential of MP

**Fig. 4:**
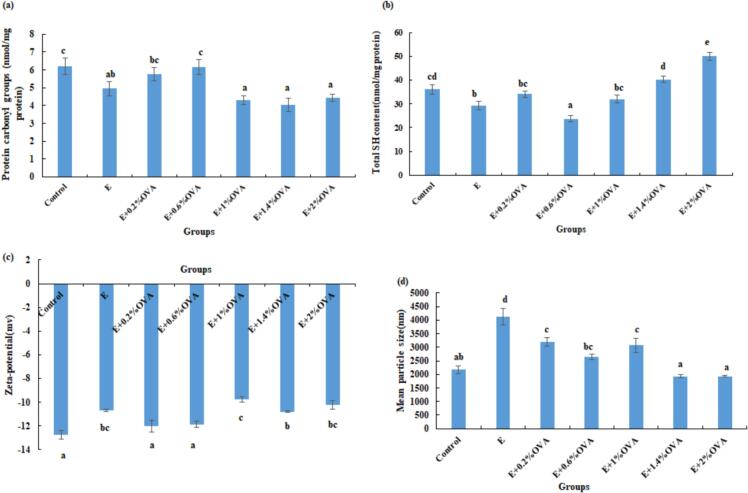
Changes in (a) protein carbonyl groups, (b) total SH content, (c) Zeta- potential and (d) mean particle size of MP fractions in all group beef. Abbreviations: Control, the untreated group; E, absolute ethanol; OVA, ovalbumin. Values are means ± SE of three replicates measurements. SH, sulfhydryl. Error bars represent positive standard errors of the mean. Different lowercase letters (a-f) donate significant difference in these treatment group samples (*P* < 0.05). Means having no letter do not significantly differ at *P* < 0.05.

The formation of carbonyl groups and loss of SH groups are widely regarded as key indicators of protein oxidation. As shown in Fig. 4(a), the content of carbonyl groups in MP of the control, E + OVA (0.2%, 0.6%) groups was significantly (*P* < 0.05) higher than those of other 4 groups. A significant decrease in carbonyl formation was also observed in 3% plant fruit extracts treated burger patties (Jongberg, Skov, Tørngren, Skibsted, & Lund, 2011). The total SH content in MP of the control, E + OVA (1.4%, 2%) groups was significantly (*P* < 0.05) higher relative to those of the E and E + 0.6%OVA groups (Fig. 4(b)). Compared with the control, E + OVA (0.2%, 0.6%, 2%) groups might inhibit protein oxidation, which may be associated with certain aromatic amino acid residues or SH-containing amino acids(e.g., tryptophan (Trp), tyrosine (Tyr), and methionine (Met)) in OVA that counteract ethanol-related protein oxidation and other modifications (Liu, Ying, Cai, & Le, 2017; Sun et al., 2001). Zheng et al. (2025) also observed that the beef marinated in red sour soup for 60 min had a lower MP oxidation level than that of 2% NaCl marinated ones.

Notably, the MP mean particle size in the E, E + OVA (0.2%, 1%) groups was dramatically (*P* < 0.05) larger than those of the control, E + OVA (1.4%, 2%) groups (Fig. 4(c)), confirming the formation of large aggregates. However, when OVA addition exceeded 1%, protein aggregates dissociated, and smaller and denser complexes might have formed, resulting in a decrease in mean particle size. Kim et al. (2024) also reported a similar trend in pulsed electric field (PEF)-treated MP and EWP. Particularly, the mean particle size of MP in the E + OVA (0.2%–2%) groups effectively mitigated the increase in MP mean particle size induced by the E group, which might be pivotal in preserving meat quality and sustaining its desirable textural attributes according to Cheng et al. (2025).

As illustrated in Fig. 4d, the absolute *ζ* potential value of MP decreased dramatically (*P* < 0.05) in the E, E + OVA (1%, 1.4%, 2%) groups relative to those of the other 3 groups, demonstrating that the negative charge and intermolecular repulsion of MP were reduced and that hydrophobic moieties were exposed on the MP surface (Yu et al., 2024), making it easier to form larger-sized particles. This was also basically verified by the increase in the mean particle size. A similar result was found for the reduced absolute *ζ* potential in UT myosin (Sun et al., 2023).

### 3.5 Dynamic rheological properties of raw MP batter

**Fig. 5:**
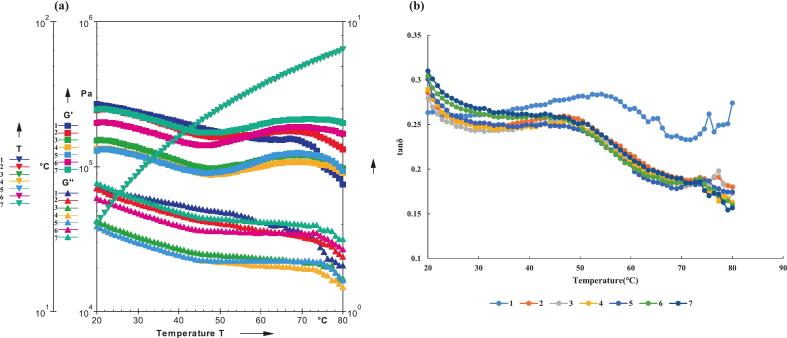
Changes in (a) the storage modulus (G'), (b) loss modulus (G"), and (c) tan δ values of MP batter in all group beef. Abbreviations: Control, the untreated group; E, absolute ethanol; OVA, ovalbumin; 1, Control; 2, E; 3, E + 0.2%OVA; 4, E + 0.6%OVA; 5, E + 1%OVA; 6, E + 1.4%OVA; 7, E + 2%OVA.

The dynamic rheological curves of raw MP batter are shown in Fig. 5(a). The G' values of all MP batter samples were larger than G" values, which might be attributed to MP denaturation, inducing the transformation of the MP batter system into a more ordered gel matrix (Kim et al., 2021). At temperatures ranging from 20 °C to 45 °C, the G' and G" values of E + OVA (0.2%, 0.6%, 1%) groups MP batter were lower than those of other 4 groups. This phenomenon might be due to the depolymerization of the myosin and weakened interaction forces (Yu et al., 2024). The G' and G" particularly in E, E + OVA(1.4%, 2%) groups presented upward trends when the temperature elevated to 50 °C, indicating that the MP batter transitioned into the gel phase, might be intensified by cross-linking of myosin tails (Yu et al., 2024). These findings also underscored the substantial interference of OVA might diminish the self-aggregation of MHC and form gels with decreased gel strength according to Nie, Xiong, and Jiang (2024).

Similarly, Zhang et al. (2025) reported an increase in the G' of MP gels treated with fibrinogen hydrolysates. The tan δ values (less than 1) indicated that the elasticity of all MP batter samples was greater than their viscosity. The tan δ values tended to decrease, especially in all group MP batter (except the control), suggesting that stronger gel networks might be formed in these groups. This observation aligned with a previous study reporting a decrease in tan δ values in porcine MP after PEF treatment (Kim et al., 2024).

### 3.6 Water-holding capacity (WHC) and textural parameters of MP gels

It can be seen from Table 2 that the WHC of MP gels in the E + 1.4%OVA group was dramatically enhanced (*P* < 0.05) compared with those in the control and E groups, demonstrating that the interaction between ethanol and OVA might improve water-retention capability of MP gels. Yu et al. (2024) noted that the WHC of MP gels was significantly enhanced with P-OVA addition. Zhou et al. (2019) also demonstrated that the WHC of CMP gels reached a maximum under 0.9% ethanol treatment. The higher WHC obtained in E + 1.4%OVA-treated MP gels might be attributed to the exposed protein residues interacting via hydrophobic interactions and hydrogen bonds, as well as the small mean particle size of the MP matrix solution (Sun et al., 2023).

**Table 2:** Changes of WHC and texture parameters of all groups of beef MP gels.

Treatments	WHC (%)	Hardness/g	Springiness	Cohesion	Gumminess/g	Chewiness/g
Control	70.18 ± 0.72^b^	2229.15 ± 29.25^ab^	0.73 ± 0.02^a^	0.53 ± 0.02^a^	1185.15 ± 27.49^a^	869.12 ± 27.82^a^
E	61.36 ± 0.90^a^	2032.16 ± 95.04^a^	0.75 ± 0.01^ab^	0.54 ± 0.02^a^	1100.43 ± 92.73^a^	829.38 ± 74.61^a^
E + 0.2%OVA	73.70 ± 3.19^bc^	2819.15 ± 38.95^c^	0.87 ± 0.01^c^	0.63 ± 0.00^c^	1784.67 ± 19.44^c^	1556.24 ± 29.54^c^
E + 0.6%OVA	73.76 ± 1.62^bc^	2485.84 ± 43.12^bc^	0.84 ± 0.01^c^	0.59 ± 0.01^b^	1475.09 ± 11.26^b^	1246.42 ± 27.40^b^
E + 1%OVA	72.73 ± 1.77^bc^	2552.98 ± 161.49^bc^	0.83 ± 0.01^c^	0.62 ± 0.01^bc^	1592.30 ± 104.18^bc^	1324.64 ± 94.93^b^
E + 1.4%OVA	76.10 ± 1.49^c^	2320.52 ± 120.07^ab^	0.82 ± 0.01^bc^	0.52 ± 0.01^a^	1199.32 ± 73.89^a^	983.86 ± 68.12^a^
E + 2%OVA	74.45 ± 1.10^bc^	2060.64 ± 174.31^a^	0.81 ± 0.04^c^	0.53 ± 0.01^a^	1086.42 ± 117.97^a^	888.02 ± 136.78^a^

WHC, water holding capacity; MP, myofibrillar protein; Control, the untreated group; E, absolute ethanol; OVA, ovalbumin; Different lowercase letters (a-f) donate significant difference in these treatment group samples (*P* < 0.05). Means having no letter do not significantly differ at *P* < 0.05.

The hardness, gumminess and chewiness values of MP gels in the E + 0.2%OVA group were significantly (*P* < 0.05) higher than those of the control, E, E + OVA(1.4%, 2%) groups, which could be ascribed to the hydrophobic and hydrogen bonding interactions among MP, ethanol, and OVA. In contrast, lower values of hardness, gumminess and chewiness were obtained in E + OVA (1.4%, 2%) group MP gels, which was attributed to excessive OVA addition (>1%). This excessive OVA addition might weaken the interaction between ethanol and MP, and resulting in depolymerization of the gel matrix. Additionally, the springiness and cohesion of MP gels treated in E + OVA (0.2%, 1%) groups were significantly (*P* < 0.05) higher than those of the control and E groups. This phenomenon might be due to the formation of hydrogen bonds, hydrophobic interactions, enhanced system stability, as well as the development of disulfide linkages and macromolecular crosslinks among MP, ethanol and OVA. Similar results were also noted by Yu et al. (2024) and Zhou et al. (2019) in MP gel treated with 0.5% P-OVA and CMP gel treated with 0.9% ethanol.

### 3.7 LF-NMR analysis of MP gels

**Fig. 6:**
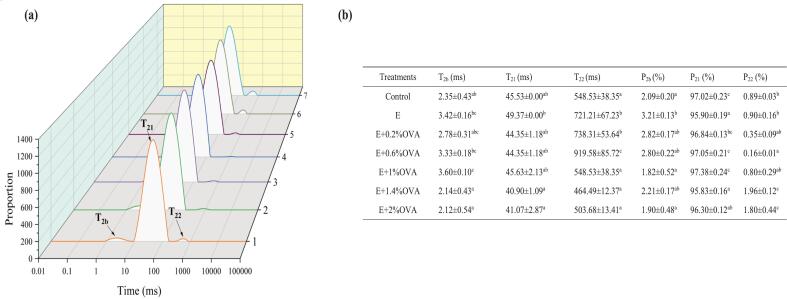
(a) Relaxation time inversion spectra of the moisture, and (b) change of relaxation time and percentage of relaxation peak area of all groups beef MP gels. Abbreviations: Control, the untreated group; E, absolute ethanol; OVA, ovalbumin; Relaxation time, T_2b_ (0–10 ms), T_21_ (10–100 ms), and T_22_ (100–1000 ms); percentages of T_2b_, T_21_, and T_22_ presented as P_2b_, P_21_, and P_22_; Different lowercase letters (a–f) donate significant difference in these treatment group samples (*P* < 0.05). Means having no letter do not significantly differ at *P* < 0.05.

Fig. 6 shows the distribution curves of T_2_ relaxation time for all group beef MP gels. Within MP gels, typically three peaks including T_2b_ (0–10 ms), T_21_ (10–100 ms) and T_22_ (100–1000 ms), were be observed, representing bound water, immobilized water, and free water, respectively. The relaxation times of these 3 types of water molecules progressively decreased (*P* < 0.05), particularly in the E + OVA (1.4%, 2%) groups relative to that of the E group. This result indicates that the combination of ethanol and OVA, rather than ethanol alone, might reduce water fluidity in MP gels, leading to a stronger interaction between water molecules and MP. These findings align with previous studies reporting the reduced relaxation times of MP gels treated with fibrinogen hydrolysates and *Toona sinensis* seed polyphenols (Wang, Zhou, Xing, Zhou, & Zhang, 2025; Zhang et al., 2025).

The peak areas corresponding to T_2b_, T_21_ and T_22_ are denoted as P_2b_, P_21_, and P_22_, respectively. Compared with those of the E, E + OVA(1.4%, 2%) groups, significantly higher P_21_ values were found in the control, E + OVA(0.2%, 0.6%,1%) group MP gels (*P* < 0.05). An apparent rise in the P_22_ was found in E + OVA (1.4%, 2%) group MP gels (*P* < 0.05). However, this trend was not clearly reflected in the WHC data, but was largely dependent on gel hardness. Excessive OVA addition (>1%) might trigger the conversion of immobilized water to free water in ethanol-treated MP gels, which could be attributed to the decreased hydrogen bonding of “intermediate water”, enhanced MP unfolding and exposure of hydrophobic groups. Nie et al. (2024) and Sun et al. (2023) have also reported similar results of water distribution in hybrid protein gels and UT-treated MP gels.

### 3.8 Secondary structure

**Fig. 7:**
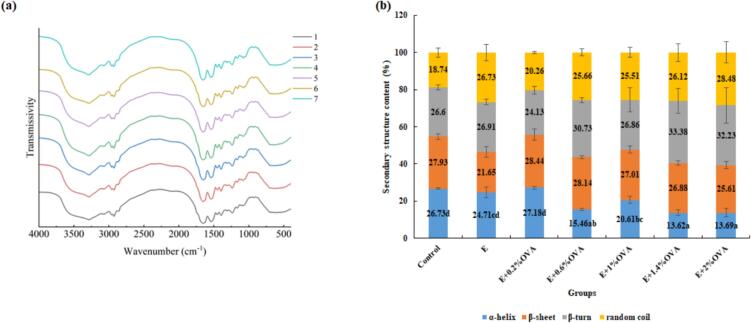
(a) FTIR spectra and (b) secondary structure content (%) of all groups beef MP gels. The samples are same as Table 1. Abbreviations: FTIR, Fourier transform infrared spectra; 1, Control; 2, E; 3, E + 0.2%OVA; 4, E + 0.6%OVA; 5, E + 1%OVA; 6, E + 1.4%OVA; 7, E + 2%OVA; Control, the untreated group; E, absolute ethanol; OVA, ovalbumin; Different lowercase letters (a–f) donate significant difference in these treatment group samples (*P* < 0.05). Means having no letter do not significantly differ at *P* < 0.05.

As demonstrated in Fig. 7(a), a slightly enhanced absorption peak was identified in E and all E + OVA groups near 3250–3000 cm^−1^, corresponding to amide A bands, This enhancement was primarily caused by stretching vibrations of N—H or O—H bonds (Liu et al., 2025). A decrease in peak intensity at 1500–2000 cm^−1^, ascribed to the C=C stretching vibration of the intramolecular hydrogen bonds (Yu et al., 2024), was observed in especially E, E + OVA(1%, 1.4%, 2%) groups. The absorption peak detected at 1115 cm^−1^, assigned to P=O stretching (Li, Sun, Ma, Jin, & Sheng, 2018), was slightly enhanced in the E, E + OVA (0.2%, 2%) group MP gels.

Changes in the secondary structure content of MP gels are shown in Fig. 7(b). Significantly higher α-helix% were obtained in control, E and E + 0.2%OVA group MP gels compared to those of the E + OVA(0.6%, 1.4%, 2%) groups (*P* < 0.05). A lower content of α-helix might induce increased disorder and flexibility of molecules, facilitating particle linkage through interaction forces during formation of MP gels (Yu et al., 2024). Similarly, a decreased α-helix% was also detected in P-OVA and fibrinogen hydrolysates-treated MP gels (Yu et al., 2024; Zhang et al., 2025). However, significantly higher contents of β-sheet (32.48%, 32.39%) were detected in the E, E + OVA (0.2%, 0.6%) groups rather than those of the E + OVA(1%, 2%) groups(31.76%, 30.98%). This finding could thus explain why the E + 0.6%OVA group exhibited superior gelling properties.

### 3.9 SEM analysis of MP-gels' structure

**Fig. 8:**
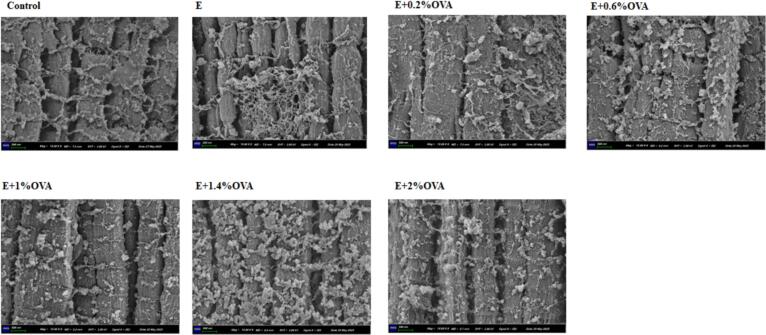
The SEM micrographs of all groups of beef MP-gels. Abbreviations: Control, the untreated group; E, absolute ethanol; OVA, ovalbumin; SEM, scanning electron microscopy.

The microstructure of all group beef MP gels at 18000× magnification is shown in Fig. 8. The surface of the control MP gels exhibited a loose net-like structure with more obvious holes Compared with the control group, the surface of E + OVA (0.2%, 0.6%, 1.4%) group MP-gels presented a more ordered and compact appearance of protein agglomeration, with some densely-packed small pores similar to a honeycomb. This phenomenon might be attributed to ethanol and OVA, which could expose hydrophobic groups on the protein surface, weaken electrostatic connection, and enhance protein interactions, aggregation and cross-linking with MP (Sun et al., 2023; Yu et al., 2024). This rigid structure effectively facilitated water binding, as evidenced by the WHC data (discussed in Table 2).

However, the microstructure of E, E + OVA (1%, 2%) group MP-gels revealed an irregular, disordered, and inhomogeneous fibrous network, with a slightly less tightly interconnected and thinner matrix, as well as larger and more brittle pores. This indicates that the gel network might be further damaged due to enhanced interactions among ethanol, OVA and MP. The gels with a coarse texture are soft and fragile and exhibit weak WHC (Nie et al., 2024). Similar results were also been observed in ethanol-treated CMP, P-OVA and UT-treated MP gels (Sun et al., 2023; Yu et al., 2024; Zhou et al., 2019).

### 3.10 Correlation analysis between SP and MP

**Fig. 9:**
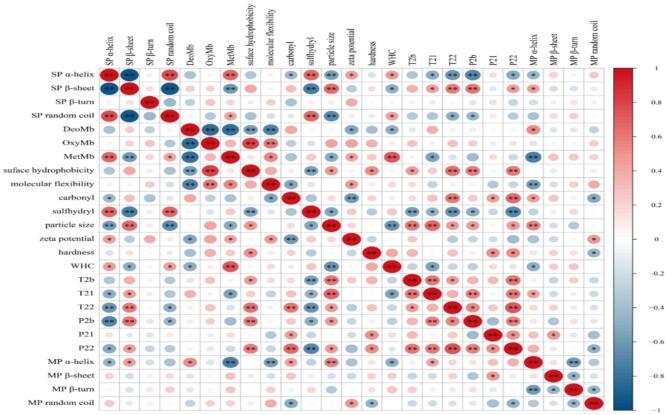
The correlation among the secondary structure, functional and other relevant indicators of beef MP and SP. *, Significant correlation at *P* < 0.05; **, Significant correlation at *P* < 0.01.

Correlation analysis was performed in order to explore the relationships among the secondary structure, functional and other relevant indicators of MP and SP. As shown in Fig. 9, the α-helix and random coil of SP exhibited significantly (*P* < 0.05) positive correlations with the contents of MetMb, sulfhydryl and WHC of MP matrix, while showing negative correlations with the content of β-sheet of SP, as well as the mean particle size, T_22_ and P_2b_ of the MP matrix. The DetMb displayed a significantly (*P* < 0.05) positive correlation with the α-helix, and negative correlations with surface hydrophobicity, molecular flexibility, zeta potential and WHC of MP matrix. The MetMb showed significantly (*P* < 0.05) positive correlations with the molecule flexibility, zeta potential and WHC, and a negative correlation with the mean particle size, T_21_ and α-helix content of the MP matrix. In addition, the mean particle size, T_21_, T_22_, P_2b_, P_22_ and α-helix content of MP matrix were negatively correlated with the α-helix, and positively correlated with the β-sheet of the SP matrix (*P* < 0.05). Treatments with ethanol and OVA resulted in the decreases in DeoMb, β-sheet of SP matrix, the content of protein carbonyl groups, zeta potential, mean particle size, α-helix, T_2b_, T_21_,T_22_, P_2b_ and P_21_ of the MP matrix, while leading to increases in content of OxyMb, MetMb, α-helix and random coil of SP matrix, hardness, WHC, surface hydrophobicity, molecular flexibility, total SH content and P_22_ of MP matrix. Combined with the results of SDS-PAGE, DSC, EIF, rheology and SEM, these findings also indicate that the addition of ethanol and OVA mainly influenced Mb forms, the secondary structures of SP and MP gels, and other relative intermolecular interactions in MP, These effects further improved the textural properties of MP gels through protein degradation, aggregation, and cross-linking procedures.

## 4 Conclusion

The present study revealed the regulatory effect of ethanol and OVA addition on the interaction characteristics, correlations of beef SP and MP matrix, and the corresponding mechanism. The influences of ethanol and various content of OVA addition on SP and MP matrices were distinct respectively. In beef SP matrix, E + OVA (≥1%) groups improved α-helix% and MetMb%, while weakened protein bands and denaturation peaks relative to that of the E + 0.2%OVA group. In beef MP matrix, the significantly higher P_22_%, carbonyl groups, mean particle size, absolute *ζ* potential, gumminess and chewiness were observed in E + OVA (≥1.4%) groups rather than those of the E + OVA (0.2%, 0.6%) groups. The slightly irregular, disordered, inhomogeneous, and less tightly interconnected fibrous gel network was detected rather in E + OVA (1%, 2%) group MP matrix. Moreover, the correlations in key properties between beef SP and MP matrix under various E + OVA treatments have been emphasized. The DeoMb%, β-sheet% in SP matrix in SP matrix negatively correlated with gel hardness, P_22_ and WHC, molecular flexibility, surface hydrophobicity and P_22_ in MP matrix. Both ethanol and various amounts of OVA addition had diversified impacts on the interaction characters of SP and MP proteins. Specifically, treatments with ethanol (5%) and OVA (≤1%) can be beneficial for future meat processing with relatively mild protein denaturation, and structure changes, and enhanced gel-related properties. Further research is still needed to reveal the mechanism by which ethanol and OVA affect the structure and interaction characteristics of meat proteins during actual processing.

## CRediT authorship contribution statement

**Yan Dai:** Writing – review & editing, Writing – original draft, Project administration, Methodology, Investigation, Funding acquisition, Data curation. **Sha Yi:** Validation, Software, Formal analysis. **Zhen Lu:** Software, Resources, Methodology, Formal analysis. **Xiao-li Pan:** Writing – review & editing, Visualization, Validation. **Yu-xin Li:** Visualization, Validation, Software, Investigation. **Yan-yan Chen:** Software, Resources, Project administration. **Feng Huang:** Supervision. **Chun-hong Li:** Writing – review & editing, Supervision.

## Declaration of competing interest

The authors declare that they have no known competing financial interests or personal relationships that could have appeared to influence the work reported in this paper.

## Data Availability

The authors do not have permission to share data.
